# Clinical outcomes after single-level posterior lumbar interbody fusion in osteoporotic patients with or without paraspinal muscle atrophy: a retrospective study

**DOI:** 10.3389/fsurg.2026.1860852

**Published:** 2026-06-23

**Authors:** Yifang Shi, Jialin Wang, Zongmian Song, Miaoheng Yan, Longyu Li, Hao Han, Hongjian Liu, Songfeng Chen

**Affiliations:** 1 Department of Orthopedics, The First Affiliated Hospital of Zhengzhou University, Zhengzhou, China; 2 Clinical Systems Biology Laboratories, Translational Medicine Center, The First Affiliated Hospital of Zhengzhou University, Zhengzhou, China; 3 Department of Basic and Translational Sciences, School of Dental Medicine, University of Pennsylvania, Philadelphia, PA, United States

**Keywords:** fat infiltration, lumbar fusion, osteoporosis, paraspinal muscles, surgical outcomes

## Abstract

**Background:**

Lumbar disc herniation (LDH) is a major cause of low back pain and disability. In osteoporotic patients undergoing posterior lumbar interbody fusion (PLIF), postoperative recovery may be influenced not only by impaired bone quality but also by paraspinal muscle degeneration. However, the prognostic value of paraspinal muscle atrophy (PMA) in this population remains unclear. This study aimed to evaluate the impact of concomitant PMA on postoperative outcomes in osteoporotic patients with LDH treated with PLIF.

**Methods:**

According to inclusion and exclusion criteria, a retrospective study was performed on LDH patients who underwent single-level posterior lumbar interbody fusion (PLIF) surgery from January 2021 to January 2025. PMA was assessed using functional cross-sectional area (FCSA) and fat infiltration (FI) as quantitative imaging indicators, and the Oswestry Disability Index (ODI), Visual Analog Scale (VAS), and SF-36 were used to evaluate surgical outcomes. In addition to these, the duration of symptoms, body mass index (BMI), bone mineral density (BMD), blood loss, and hospital stay after surgery were measured for all patients.

**Results:**

A total of 142 osteoporotic patients with LDH treated with PLIF were included (OP, *n* = 90; OP + PMA, *n* = 52). The OP + PMA group exhibited significantly greater paraspinal muscle degeneration, with lower FCSA and higher FI% of the bilateral posterolateral lumbar muscle groups. Although both groups improved after surgery, the OP + PMA group had persistently higher VAS scores throughout follow-up, worse ODI at 6 and 12 months, and smaller ODI improvement from baseline. SF-36 Bodily Pain scores were significantly lower in the OP + PMA group at all time points, while SF-36 Physical Function scores were significantly lower at 6 and 12 months. In addition, improvements in both SF-36 domains from baseline were significantly smaller in the OP + PMA group than in the OP group.

**Conclusion:**

Among osteoporotic patients with LDH undergoing PLIF, concomitant PMA was associated with poorer postoperative recovery and worse functional prognosis.

## Introduction

Lumbar disc herniation (LDH) is a common degenerative spinal disorder and one of the leading causes of low back pain and lumbar radiculopathy. Compression and irritation of the affected nerve root may result in persistent leg pain, sensory disturbance, motor weakness, and functional disability, thereby substantially impairing patients’ quality of life (1). As population aging accelerates, the incidence and clinical burden of LDH continue to rise, posing substantial challenges to both healthcare systems and society (2). For selected patients with refractory symptoms or progressive neurological impairment, PLIF may be an effective surgical option to alleviate pain, restore spinal alignment and stability, and improve functional capacity.

Among the available surgical techniques, posterior lumbar interbody fusion (PLIF) is widely used for the management of LDH because it enables direct neural decompression, reconstruction of disc height, and biomechanical stabilization of the affected segment (3). Nevertheless, postoperative outcomes following PLIF remain heterogeneous. While many patients achieve satisfactory symptom relief and functional recovery (4), others experience persistent pain, delayed rehabilitation, or surgery-related complications. This variability suggests that, beyond operative technique itself, patient-specific biological and structural factors may substantially influence surgical prognosis (5).

Osteoporosis (OP) is one such factor that has attracted considerable attention in spine surgery. Poor bone quality may impair pedicle screw fixation, compromise fusion stability (6), and predispose patients to instrumentation-related complications, including cage subsidence, loosening, and nonunion (7). Meanwhile, the importance of paraspinal muscle condition in spinal degeneration and postoperative recovery has been increasingly recognized. As a major dynamic stabilizer of the lumbar spine, the paraspinal musculature plays a fundamental role in maintaining sagittal balance and segmental control (8). Paraspinal muscle atrophy (PMA), typically manifested as reduced functional cross-sectional area (FCSA) and increased fat infiltration (FI), has been linked to chronic low back pain, reduced physical performance, and spinal instability. Such muscular degeneration may further limit postoperative recovery and compromise long-term clinical outcomes (9, 10).

Although previous studies have separately examined the influence of OP or PMA in lumbar degenerative disorders, the interactive or cumulative effect of these two conditions on outcomes after PLIF has yet to be clearly defined. Moreover, prognostic tools that jointly integrate skeletal and muscular status remain scarce, limiting their utility in preoperative decision-making and risk stratification.

Therefore, this retrospective study aimed to evaluate and compare the clinical outcomes after single-level PLIF in osteoporotic patients with or without PMA. By assessing back muscle degeneration, we sought to elucidate its specific impact on postoperative pain, functional recovery, and long-term quality of life, thereby providing valuable clinical evidence for preoperative risk stratification.

## Materials and methods

### Subjects and ethics approval

This retrospective study was based on patients’ medical records from January 2021 to January 2025 at the First Affiliated Hospital of Zhengzhou University. It was approved by the Ethics Committee of The First Affiliated Hospital of Zhengzhou University (2023-KY-0346-002).

### Inclusion and exclusion criteria

The inclusion criteria were as follows: (i) age ≥ 50 years old; (ii) MRI-confirmed single-level LDH; (iii) DXA-confirmed osteoporosis (T-score ≤ −2.5 at lumbar spine or femoral neck) (iv) met the indications for PLIF and had failed conservative treatment for >6 months; (v) Complete clinical data and complete postoperative follow-up for 12 months.

The presence of any of the following conditions excluded from the study: (i) previous lumbar spine surgery or the presence of lumbar spine tumor, infection, fracture, or history of ankylosing spondylitis; (ii) patients with combined congenital spinal deformities; (iii) patients diagnosed with secondary OP; (iv) patients with combined cauda equina syndrome (CES); (v) follow-up period was <1 year or refuse to review on time.

**Figure 1:**
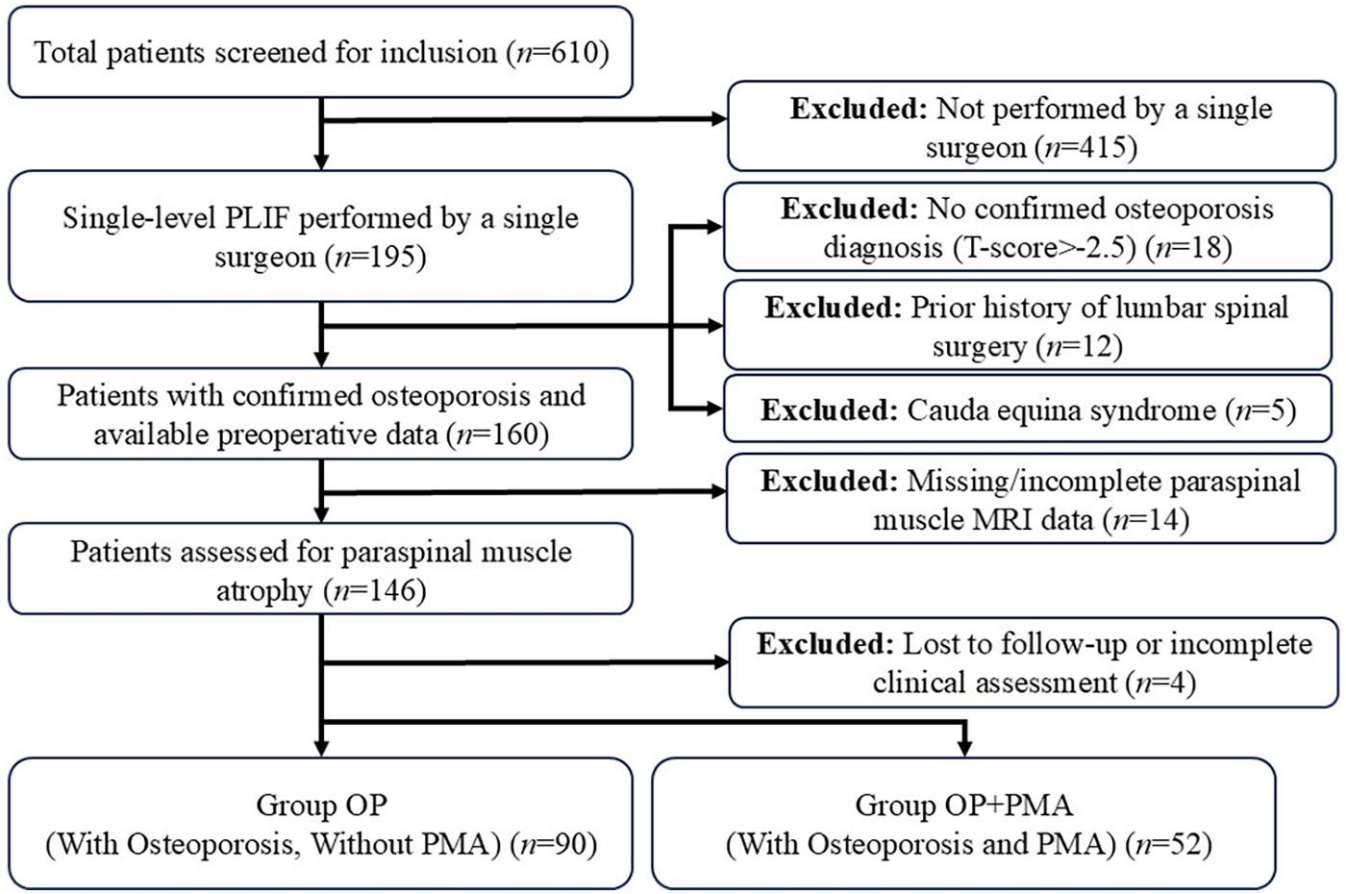
STROBE flowchart delineating patient screening and study allocation.

All operations were performed by a single surgical team with over 10 years of experience in spine surgery, and all decompression operations were performed by the same surgeon. All patients underwent conventional open single-level PLIF under general anesthesia. Through a standard posterior approach, decompression of the affected level, discectomy, endplate preparation, cage implantation, and pedicle screw fixation were performed. Perioperative management followed routine institutional practice, including early discontinuation of antibiotics, early drain removal, and early mobilization after surgery. A thoracolumbar brace was routinely used for protection for 3 months postoperatively, and all patients received routine rehabilitation treatment. Bone health management was individualized according to the severity of osteoporosis and included vitamin D and calcium supplementation, with calcitonin or denosumab administered when indicated. Postoperative fusion status was assessed using plain radiographs at the routine follow-up time points. Radiographic fusion was considered present when there was evidence of bony bridging and trabecular remodeling across the interbody space without obvious radiolucency around the cage or screws, implant failure, or false motion. The detailed patient screening, exclusion pathways, and final cohort allocation in accordance with the STROBE guidelines are systematically illustrated in Figure 1.

A total of 610 patients who underwent single-level PLIF were initially evaluated. To eliminate technical confounding and ensure clinical homogeneity, cases not performed by the same senior surgeon (*n* = 415) were excluded. After subsequent exclusions based on strict eligibility criteria—including the absence of a confirmed osteoporosis diagnosis (T-score > −2.5, *n* = 18), prior lumbar surgery history (*n* = 12), and cauda equina syndrome (*n* = 5)—160 patients remained. Following further attrition due to missing paraspinal muscle MRI data (*n* = 14) and loss to follow-up (*n* = 4), the final 142 patients were allocated into two parallel cohorts according to their preoperative paraspinal muscle status: Group OP (Osteoporosis without PMA, *n* = 90) and Group OP + PMA (Osteoporosis with concomitant PMA, *n* = 52).

### Diagnosis of OP

BMD of the lumbar spine (L1 to L4) and the left femoral neck of the patients was measured. T-values were automatically calculated using a Prodigy Lunar type DXA bone densitometer manufactured by General Electric (GE). The diagnosis of OP was carried out according to the evaluation criteria established by the World Health Organization (11). Osteoporosis was defined as a T-score ≤ −2.5 at either the lumbar spine (L1–L4) or the left femoral neck.

### Measurement of PMA

**Figure 2:**
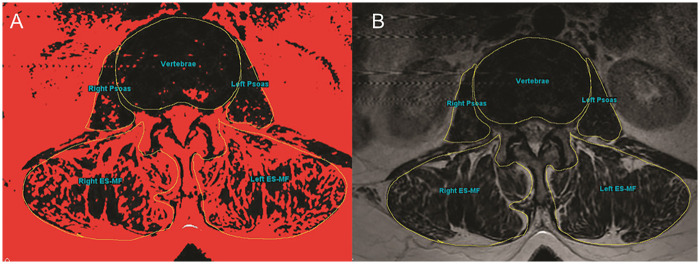
Representative MRI-based measurement of paraspinal muscle morphology and fatty infiltration at the L4 level. **(A)** Threshold-based segmented axial T2-weighted MRI image used to quantify fatty infiltration. **(B)** Original axial T2-weighted MRI image with manually outlined regions of interest (ROIs). The vertebral body and bilateral erector spinae–multifidus muscle groups (ES–MF) were included in the quantitative analysis. Bilateral psoas muscles are shown for anatomical orientation only. Cross-sectional area (CSA), functional cross-sectional area (FCSA), and fat infiltration (FI) were measured using ImageJ.

All patients were scanned using an Ingenia 3.0 T MRI imaging system from PHILIPS, with the MRI scanning axis always parallel to the upper and lower endplates of the lumbar vertebrae. Three T2-weighted lumbar cross-sectional images were taken for each lumbar vertebral segment, and images were analyzed using ImageJ for the bilateral lumbar paraspinal muscles and vertebral bodies. Based on previous studies, the muscles at the level of the L4 superior endplate were selected for analysis (12, 13). However, because it is difficult to accurately distinguish between the multifidus (MF) and erector spinae (ES) muscles in the images and because both muscles are dorsal extensors that maintain the balance of the sagittal and coronal planes of the spine, the multifidus and erector spinae muscles have been considered together as the posterior lumbar posterolateral muscle group (MF-ES). Therefore, the present study analyzed them as the posterior lumbar posterolateral muscle group (14). Three spine surgeons, who did not know the patients’ information, used ImageJ to perform measurements on the L4 lower vertebral endplate levels of all patients with bilateral ES-MFs set as the region of interest (ROI) to accurately quantify the muscle cross-sectional area (CSA) of the posterior paraspinal muscles on both sides. Fat infiltration area (FIA) was determined bilaterally using the automatic thresholding technique in Image J, and the percentage of fat infiltration (FI%) was also calculated. Fatty infiltration was quantified using threshold-based measurements in ImageJ, and severe fatty degeneration was interpreted with reference to the Goutallier classification system (14, 15). All five ROI selections and fat infiltration are shown in Figure 2. The paraspinal muscles’ functional cross-sectional area (FSCA) was used instead of the CSA for the calculations to minimize the interference of body size differences in the measurements. The final average of the results from the three spine surgeons was selected, and a mean FI% greater than 50% was chosen as the cutoff value for PMA (16). In addition to testing reliability, we also tested the consistency and trustworthiness of the index by repeated sampling, again randomly selecting all muscle parameters in 20 patients and having two observers perform the measurements independently of each other. After three weeks, one of the observers repeated the exact measurement. The intra- and inter-observer intraclass correlation coefficients were both >0.75, indicating good reliability (17).

Based on their OP and PMA diagnoses, patients were divided into two groups: Group OP and Group OP + PMA.

### Statistical analysis

The data were analyzed using SPSS software version 26.0 (IBM Inc., Chicago, IL). Continuous variables with an approximately normal distribution are expressed as the mean and standard deviation (SD). Between-group comparisons were performed using Student's t-test or Mann–Whitney U test, as appropriate. Categorical variables were compared using the chi-square test or Fisher's exact test. A *P* value <0.05 was considered statistically significant.

## Results

### General results

Strictly based on the inclusion and exclusion criteria, a total of 142 cases were included in this study. The mean age was 64.90 ± 5.84 years. 116 patients (81.7%) were females, and 26 (18.3%) were males. 90 patients were placed in Group OP, and 52 in Group OP + PMA. Decompression levels ranged from L3/4 to L4/5, with a single level decompressed. There were no significant between-group differences in sex distribution, age, symptom duration, BMI, T-score, postoperative stay, or blood loss (Table 1 and all *P* > 0.05).

**Table 1:** Baseline demographic and perioperative characteristics of osteoporotic patients with LDH undergoing single-level PLIF.

Variable	All patients （*n* = 142)	Group OP （*n* = 90)	Group OP + PMA （*n* = 52)	*P*
Gender(M/F)	26:116	14:76	12:40	0.27
Age(years)	64.90 ± 5.84	65.40 ± 5.98	64.04 ± 5.60	0.18
Duration(months)	19.63 ± 21.24	18.11 ± 22.12	22.25 ± 19.78	0.26
BMI(kg/m^2^)	25.06 ± 4.38	24.66 ± 4.64	25.74 ± 3.88	0.32
T-score	−3.15 ± 0.50	−3.21 ± 0.51	−3.05 ± 0.48	0.07
Postoperative stay(days)	9.23 ± 3.71	9.67 ± 3.56	8.46 ± 3.90	0.07
Blood loss(mL)	227.42 ± 116.48	226.00 ± 142.93	228.85 ± 116.42	0.89

Values are presented as mean ± SD or *n* (%). *P* values indicate between-group comparisons between Group OP and Group OP + PMA. BMI, body mass index.

### PMA results

We measured FCSA and FI% of bilateral ES-MF in all patients according to the PMA measurements described above and organized the results in the following figure.

**Figure 3:**
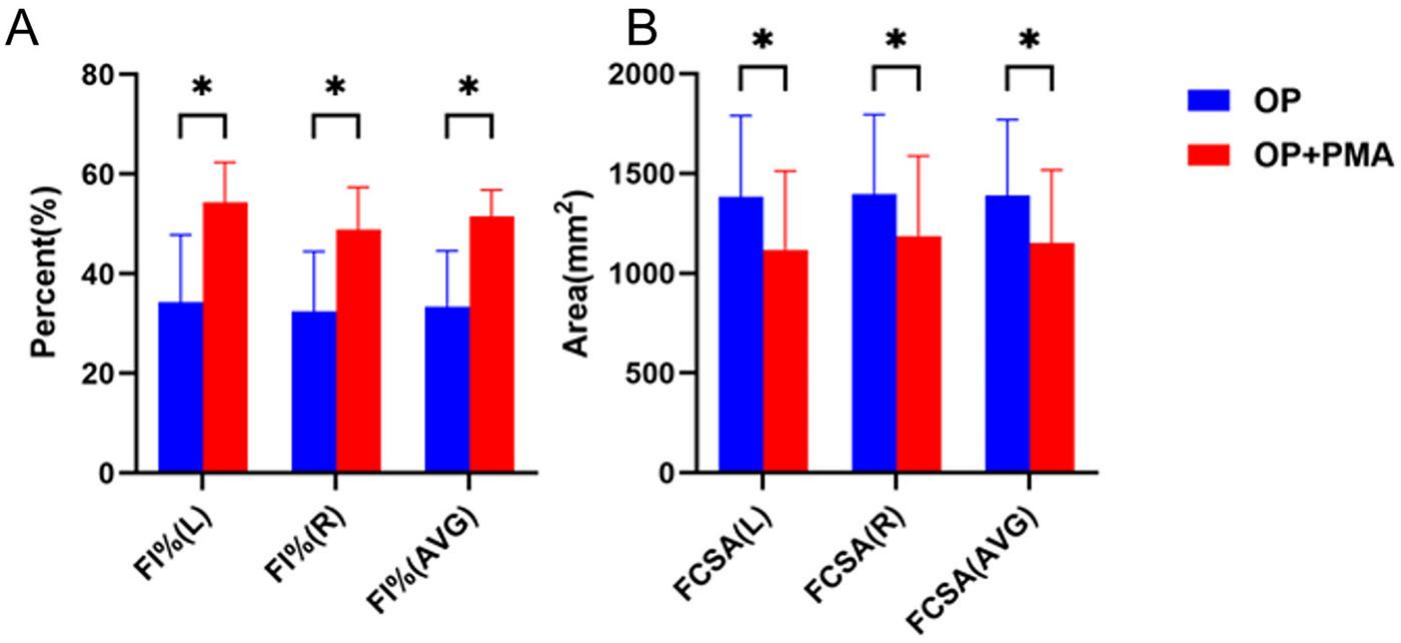
Quantitative comparison of paraspinal muscle degeneration between the OP and OP + PMA groups at the L4 level. **(A)** Fat infiltration percentage (FI%) of the bilateral erector spinae–multifidus muscle groups (ES–MF). **(B)** Functional cross-sectional area (FCSA) of the bilateral ES–MF. Higher FI% and lower FCSA indicate more severe paraspinal muscle degeneration. L, left; R, right; AVG, bilateral average. Data are presented as mean ± SD. *P < 0.05 for between-group comparisons.

**Figure 4:**
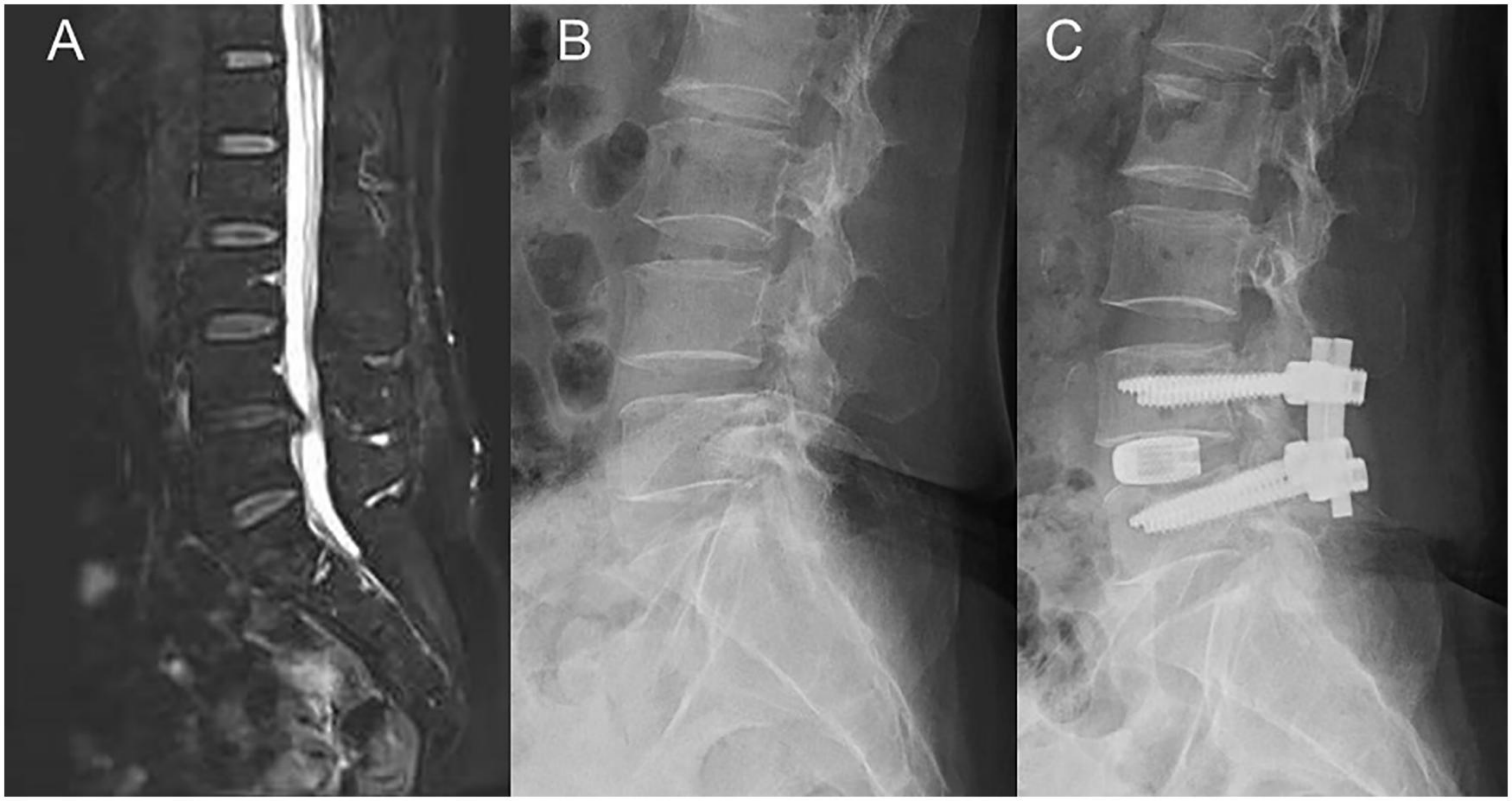
Representative imaging findings of a patient with single-level lumbar disc herniation treated with PLIF. **(A)** Preoperative sagittal T2-weighted MRI showing single-level lumbar disc herniation. **(B)** Preoperative lateral lumbar radiograph. **(C)** Postoperative lateral lumbar radiograph showing single-level posterior lumbar interbody fusion with cage implantation and pedicle screw fixation. This figure is presented for illustrative purposes only and was not included in the quantitative group comparison.

As shown in Figure 3, the OP + PMA group demonstrated significantly greater paraspinal muscle degeneration than the OP group. Specifically, FI% was significantly increased on both the left and right sides, as well as in the bilateral average, in patients with concomitant PMA. Conversely, FCSA was significantly lower in the OP + PMA group than in the OP group across all measured regions. These data indicate that patients in the OP + PMA group had more severe paraspinal muscle atrophy and fatty degeneration than those in the OP group. Representative preoperative MRI, preoperative radiograph, and postoperative radiograph of a patient with single-level LDH treated with PLIF are shown in Figure 4.

### Clinical outcomes

Clinical outcomes, including VAS, ODI, and SF-36 scores, were compared between groups at 3, 6, and 12 months postoperatively (Table 2). Both groups showed postoperative improvement in pain, disability, and health-related quality of life; however, recovery was consistently less favorable in the OP + PMA group. VAS scores were significantly higher in the OP + PMA group than in the OP group at baseline, 3 months, 6 months, and 12 months (all *P* *<* 0.05), indicating persistently greater pain in patients with concomitant PMA. Although baseline ODI and 3-month ODI did not differ significantly between groups, the OP + PMA group had significantly higher ODI scores at 6 and 12 months and a significantly smaller overall improvement from baseline (all *P* < 0.001), suggesting delayed and incomplete functional recovery. Similarly, SF-36 BP scores were significantly lower in the OP + PMA group at all time points, whereas SF-36 PF scores became significantly lower at 6 and 12 months, despite no significant difference at baseline or at 3 months. In addition, improvements in both SF-36 BP and PF from baseline were significantly smaller in the OP + PMA group than in the OP group (both *P* < 0.001). Overall, these findings suggest that the coexistence of PMA in osteoporotic patients is associated with greater postoperative pain burden, inferior functional improvement, and poorer recovery in quality-of-life measures after surgery.

**Table 2:** VAS, ODI, and SF-36 scores at baseline and during follow-up in the OP and OP + PMA groups.

Variable	Group OP	Group OP + PMA	*P*
VAS			
Baseline	4.76 ± 0.71	5.65 ± 0.69	<0.001
3months	3.00 ± 0.90	3.81 ± 0.94	<0.001
6months	2.18 ± 0.72	2.65 ± 0.69	0.008
12months	2.09 ± 0.67	2.50 ± 0.71	0.020
*Δ*VAS	2.67 ± 0.80	3.15 ± 0.94	0.002
ODI			
Baseline	50.84 ± 7.38	49.62 ± 6.41	0.332
3months	25.16 ± 3.43	25.08 ± 3.03	0.889
6months	15.11 ± 3.16	23.65 ± 3.35	<0.001
12months	9.29 ± 3.47	17.65 ± 3.24	<0.001
*Δ*ODI	41.56 ± 7.99	31.96 ± 7.72	<0.001
SF-36 BP			
Baseline	31.84 ± 10.63	25.96 ± 9.84	0.002
3months	52.63 ± 11.84	40.82 ± 10.63	<0.001
6months	63.92 ± 10.42	51.37 ± 9.96	<0.001
12months	68.43 ± 9.73	56.24 ± 9.53	<0.001
*Δ*BP	36.59 ± 7.99	30.28 ± 10.82	<0.001
SF-36 PF			
Baseline	34.52 ± 11.2	33.18 ± 10.72	0.490
3months	49.33 ± 10.7	45.74 ± 10.28	0.060
6months	58.73 ± 10.1	50.46 ± 9.71	<0.001
12months	63.22 ± 9.50	53.88 ± 9.34	<0.001
*Δ*PF	28.70 ± 7.99	20.70 ± 9.64	<0.001

Values are presented as mean ± SD. *P* values indicate between-group comparisons at each time point. Higher VAS and ODI scores indicate worse pain and disability, whereas higher SF-36 BP and PF scores indicate better health-related quality of life. *Δ*VAS and *Δ*ODI were calculated as baseline minus 12-month scores; *Δ*BP and *Δ*PF were calculated as 12-month minus baseline scores. BP, bodily pain; PF, physical function.

Multiple linear regression analyses were performed to identify independent factors associated with postoperative VAS and ODI scores, and the results are presented in Table 3. For VAS scores, the overall regression model was statistically significant, F(7,134) = 3.412, *P* = 0.0022, explaining 15.13% of the variance in VAS scores. After adjustment for age, sex, BMI category and BMD, PMA remained independently associated with higher VAS scores compared with non-PMA patients (*β*=0.397, *P* = 0.0008). BMD was negatively associated with VAS scores (*β*=−0.357, *P* = 0.0012), indicating that lower BMD was associated with greater pain intensity. Age, sex and BMI category were not significant predictors of VAS scores. For ODI scores, the overall regression model was also statistically significant, F(7,134) = 30.41, *P* < 0.0001, explaining 61.37% of the variance in ODI scores. After adjustment for age, sex, BMI category and BMD, PMA was independently associated with higher ODI scores (*β*=8.400, *P* < 0.0001). In contrast, age, sex, BMI category and BMD were not significantly associated with ODI scores in the adjusted model. These findings suggest that PMA was independently associated with both greater postoperative pain and worse functional disability, whereas lower BMD was mainly associated with higher pain intensity.

**Table 3:** Multivariable linear regression analysis of factors associated with final follow-up functional outcomes.

Outcome	Variable	Estimate	SE	95% CI	t value	*P* value
VAS	PMA	0.39	0.11	0.167–0.62	3.42	<0.001
	Age	0.08	0.11	−0.13–0.30	0.77	0.43
	Gender	−0.04	0.13	−0.30–0.22	0.29	0.76
	BMI category	—	—	—	—	0.86
	Underweight	0.05	0.25	−0.44–0.55	0.20	0.83
	Overweight	0.11	0.13	−0.15–0.37	0.84	0.39
	Obesity	0.08	0.15	−0.22–0.39	0.54	0.58
	BMD	−0.35	0.10	(−0.57)–(−0.14)	3.30	0.001
ODI	PMA	8.40	0.60	7.21–9.58	14.04	<0.001
	Age	0.20	0.57	−0.92–1.33	0.35	0.72
	Gender	−0.39	0.70	−1.77–0.99	0.55	0.58
	BMI category	—	—	—	—	0.51
	Underweight	1.93	1.31	−0.65–4.51	1.47	0.14
	Overweight	0.32	0.69	−1.03–1.68	0.46	0.64
	Obesity	0.62	0.80	−0.96–2.20	0.77	0.44
	BMD	0.16	0.56	−0.94–1.26	0.28	0.78

Normal BMI, defined as 18.5–23.9 kg/m^2^, was used as the reference category in both regression models. BMI categories were defined as underweight, <18.5 kg/m^2^; normal, 18.5–23.9 kg/m^2^; overweight, 24.0–27.9 kg/m^2^; and obesity, ≥28.0 kg/m^2^. PMA, age and gender were entered as categorical variables, whereas BMD was entered as a continuous variable.

### Postoperative complications

A summary of postoperative complications and their subsequent management across the two groups is presented in Table 4. Overall, the incidence of complications was 3.3% (3/90) in the Group OP and 7.7% (4/52) in the Group OP + PMA (Table 4).

**Table 4:** Postoperative complications, management, and outcomes in the OP and OP + PMA groups.

Complication	*n* (%)	Management and outcome
Group OP	3 (3.3)	
Epidural hematoma	1 (1.1)	Relief after emergency surgery.
Cage subsidence	2 (2.2)	A thoracolumbar brace was used for protection until bony fusion is visible on CT
Group OP + PMA	4 (7.7)	
Nerve root injury	2 (3.8)	Symptoms were resolved after 3 days of neurotrophic therapy.
Cage subsidence	2 (3.8)	A thoracolumbar brace was used for protection until bony fusion is visible on CT

Values are presented as *n* (%). Percentages were calculated within each group (Group OP, *n* = 90; Group OP + PMA, *n* = 52). This table summarizes the type of postoperative complication, corresponding management, and short-term outcome in each group. No severe long-term neurological deficits or construct failures occurred in either group.

In the Group OP, one patient (1.1%) experienced an epidural hematoma, which was successfully managed with emergency surgical decompression, leading to complete symptom relief. Additionally, two patients (2.2%) exhibited cage subsidence during the follow-up period; these patients were managed conservatively with a thoracolumbar brace for protection until solid bony fusion was confirmed via CT scans.

In the Group OP + PMA, two patients (3.8%) suffered from nerve root injury, with symptoms fully resolving after 3 days of neurotrophic therapy. Cage subsidence was observed in another two patients (3.8%) in this group. Similar to the Group OP, they were treated with a thoracolumbar brace until radiographic bony fusion was clearly visible on CT.

No severe long-term neurological deficits or construct failures occurred in either group.

## Discussion

The present study demonstrated that, among osteoporotic patients with LDH undergoing PLIF, concomitant paraspinal muscle atrophy was associated with less favorable postoperative recovery. Compared with the OP group, the OP + PMA group showed more severe paraspinal muscle degeneration, as reflected by lower functional cross-sectional area and greater fatty infiltration of the bilateral posterolateral lumbar muscle groups. In addition, these patients had persistently higher postoperative pain scores, worse disability at mid- and late-term follow-up, and less improvement in health-related quality of life. These findings suggest that PMA may represent an additional adverse prognostic factor in osteoporotic patients treated with PLIF.

One possible explanation is that osteoporosis and paraspinal muscle degeneration may impair postoperative recovery through distinct but complementary biomechanical mechanisms. In PLIF procedures, adequate bone quality contributes to fixation stability and successful fusion. Reduced bone mineral density weakens vertebral structural strength and may increase the risks of screw loosening, cage subsidence, and segmental instability (18). At the same time, degeneration of the posterior paraspinal muscles reduces dynamic spinal support, impairs movement control, and may delay postoperative rehabilitation. The coexistence of these abnormalities may therefore place patients at a dual mechanical disadvantage (19), contributing to persistent pain and incomplete functional recovery (20). In addition to worse clinical recovery, the OP + PMA group showed a numerically higher rate of postoperative complications than the OP group (7.7% vs. 3.3%). Although no severe long-term neurological deficits or construct failures occurred, this finding may still suggest a less favorable postoperative mechanical environment in patients with concomitant PMA. Reduced paraspinal muscle mass and increased fatty infiltration may weaken dynamic spinal support, impair load sharing across the operated segment, and thereby increase susceptibility to complications such as cage subsidence. In our cohort, cage subsidence occurred in both groups but was proportionally more frequent in the OP + PMA group. Nevertheless, because the absolute number of complications was small, these results should be interpreted cautiously and require further confirmation in larger studies with standardized radiographic assessment. Although formal fusion quality could not be systematically graded because postoperative CT was not routinely available in all patients, the coexistence of poor bone quality and paraspinal muscle degeneration may still have affected the fusion-related mechanical environment. Reduced muscular support may impair segmental load sharing and increase stress concentration at the operated level, thereby potentially predisposing patients to cage subsidence, delayed structural adaptation, and less favorable long-term recovery.

An important observation in the present study is that the influence of PMA was more evident in mid- and long-term outcomes than in early postoperative function. Although VAS scores differed between groups throughout follow-up, the between-group differences in ODI and SF-36 PF became more pronounced at 6 and 12 months. This pattern suggests that PMA may have a greater effect on long-term functional recovery than on short-term symptom relief. While surgical decompression can reduce pain in the early postoperative period, restoration of physical function likely depends increasingly on muscular support, spinal stability, and rehabilitation capacity over time.

From a clinical perspective, these findings highlight the importance of preoperative assessment of paraspinal muscle status in addition to bone quality (21). Osteoporotic patients with concomitant PMA may represent a high-risk subgroup requiring more individualized perioperative management (22, 23). In such patients, postoperative care should extend beyond routine follow-up and may benefit from a more comprehensive strategy, including bone health optimization, targeted rehabilitation, and structured paraspinal muscle training (24).

Several limitations should be acknowledged. First, this was a retrospective single-center study, which may have introduced selection bias and limited the generalizability of the findings. Second, PMA was assessed using imaging-derived parameters rather than direct measures of muscle strength or physical performance; therefore, our findings mainly reflect local structural muscle degeneration. Third, potentially relevant confounders, including rehabilitation adherence, nutritional status, and physical activity, could not be fully controlled. Furthermore, because postoperative CT was not routinely available for all patients at a standardized follow-up time point, formal fusion status could not be systematically assessed or compared between groups. Although MIS-TLIF is now standard due to reduced trauma, open PLIF was used because MIS-TLIF was not yet routine at our center during the early phase of this retrospective cohort. To guarantee safety and treatment consistency for these osteoporotic patients, we selected PLIF based on our mature experience. Future research will focus on MIS-TLIF outcomes. Despite these limitations, the present study indicates that paraspinal muscle atrophy is associated with poorer postoperative recovery in osteoporotic patients with LDH undergoing PLIF, and supports the clinical value of incorporating paraspinal muscle evaluation into preoperative risk assessment.

## Conclusion

In conclusion, concomitant paraspinal muscle atrophy in osteoporotic patients undergoing PLIF is a critical risk factor for suboptimal postoperative recovery and persistent pain. Routine preoperative evaluation of paraspinal musculature is thus recommended to guide risk stratification and individualized clinical decision-making.

## Data Availability

The original contributions presented in the study are included in the article/Supplementary Material, further inquiries can be directed to the corresponding authors.
